# Surface lattice resonances for beaming and outcoupling green ***μ*** LEDs emission

**DOI:** 10.1515/nanoph-2023-0257

**Published:** 2023-08-04

**Authors:** Mohamed S. Abdelkhalik, Aleksandr Vaskin, Toni López, Anton Matthijs Berghuis, Aimi Abass, Jaime Gómez Rivas

**Affiliations:** Department of Applied Physics and Science Education, and Eindhoven Hendrik Casimir Institute, P.O. Box 513, 5600 MB Eindhoven, The Netherlands; Lumileds Germany GmbH, D-52068 Aachen, Germany

**Keywords:** *μ*LEDs, light beaming, plasmonics, SLR

## Abstract

Light-Emitting Diodes (LEDs) exhibit a typical Lambertian emission, raising the need for secondary optics to tailor their emission depending on specific applications. Here, we introduce plasmonic metasurfaces to InGaN green emitting quantum wells for LEDs to control their far-field emission directionality and enhance the collection efficiency. The proposed mechanism is based on surface lattice resonances (SLRs) and relies on the near-field coupling between the InGaN multiple quantum wells (MQWs) and periodic arrays of aluminum (Al) nanodisks. Fourier microscopy measurements reveal that the angular photoluminescence emission pattern depends on the lattice constant of the metasurfaces. We demonstrate that integrating Al metasurfaces in LED wafers can enhance the collected outcoupled light intensity by a factor of 5 compared to the same sample without metasurfaces. We have also performed numerical calculations of the far-field emission based on the reciprocity principle and obtained a very good agreement with the experimental data. The proposed approach controls the emission directionality without the need for secondary optics and it does not require post-etching of the GaN, which makes it a potential candidate to control and enhance the generated light from micro-LEDs.

## 1 Introduction

Micro-light-emitting diodes (*μ*LEDs) for display technology attracting broad interest due to their unique features, including improved lifetime, compact size, high brightness, high contrast, and low power consumption [1–3]. *μ*LEDs based on gallium nitride (GaN) have great potential in developing a variety of applications beyond displays, such as wireless optical communication and wearable electronics, e.g., head-mounted displays for virtual reality (VR) and augmented reality (AR) [4–6]. However, GaN-based LEDs and *μ*LEDs have low outcoupling efficiency as a result of the high-index mismatch between air and GaN: the generated light at angles larger than the critical angle for total internal reflection (*θ*_*c*_) is trapped in the device [7–10]. Volcano, moth-eye, or pyramids-shaped patterned sapphire substrates can be used to enhance the outcoupling efficiency in high-power LEDs [11–13]. However, the surface texture approaches are not compatible with *μ*LEDs as they have bigger sizes than *μ*LEDs ( <50 μm). Therefore, mesa sidewalls edges are used for enhancing the light outcoupling of *μ*LEDs devices with different diameters from 2 to 10 μm, which relies on post-growth etching through the GaN layer [14–19]. These etched sidewalls act as micro-reflectors for the generated light, leading to an enhanced light outcoupling [14, 15]. This approach leads also to sidewall defects that increase the non-radiative recombination, and cause a significant reduction in the internal quantum efficiency of the device [20–22].

A promising approach to enhance the light outcoupling efficiency and minimize the non-radiative recombination is engineering the photonic environment of the quantum wells [23]. Structured thin films of metals supporting surface plasmon polaritons have been proposed for this outcoupling [24, 25]. However, the losses remain high and light outcoupling is limited to quantum wells in close proximity to the metal. Metasurfaces provide an alternative way of engineering the surroundings of the quantum wells to significantly increase the outcoupling efficiency and control the emission directionality from *μ*LEDs [8, 26–31]. The metasurfaces consist of resonant nanoparticles made of metal, such as Al, Au, or Ag, supporting localized surface plasmon resonances (LSPRs), i.e., coherent oscillations of electrons in the nanoparticles [23, 32–37]. Similar effects can be obtained with dielectrics, for example, Si or TiO_2_ supporting Mie resonances, i.e., electromagnetic resonances in the nanoparticles [30, 38–42]. Similar to thin metal films, the near field associated to localized resonances is enhanced only very close (a few nanometers) to the surface [26].

Arrays of nanoparticles may support collective plasmonic resonances known as surface lattice resonances (SLRs), which are the result of the enhanced radiative coupling between the resonances of the individual nanoparticles through in-plane diffraction orders known as Rayleigh anomalies (RAs) [41, 43–50]. A characteristic of SLRs is that they support high near-field enhancements, which are weakly confined to the individual nanoparticles. This enhancement can reach a large distance from the individual nanoparticles, enabling the coupling to distant emitters.

In this manuscript, we introduce arrays of Al metasurfaces that support SLRs at the emission wavelength of InGaN MQWs of green LEDs, i.e., 570 nm. The investigated arrays are arranged in a square lattice with varying lattice constants at a distance of 95 nm away from MQWs. This approach relies on the near-field coupling between the SLRs from the Al metasurfaces and the MQWs. The weak confinement of SLRs allows reaching a larger distance from the surface than localized resonances and surface plasmon polaritons. For commercial LEDs and micro-LEDs, the MQWs are typically located at about 100 nm from the surface. Therefore, SLRs are a promising approach for achieving near-field coupling between the metasurfaces and the MQWs. This near-field coupling introduces new degrees of freedom to reshape the far-field emission and to beam the generated light into a narrow solid angle, which is essential for several applications, as well as for improving light outcoupling.

## 2 Results and discussion

### 2.1 System description

**Figure 1:**
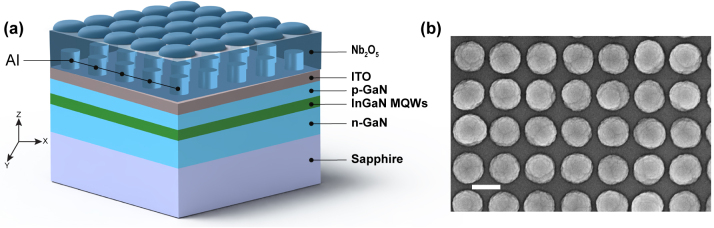
Investigated sample description. (a) Al metasurfaces placed on a GaN LED wafer and covered by a layer of Nb_2_O_5_. The laser emission (*λ* = 405 nm) illuminating the sample from the sapphire side excites the InGaN MQWs, which emit at 570 nm. (b) SEM top view of the Al nanodisks covered by Nb_2_O_5_. The scale bar corresponds to 240 nm.

We have fabricated samples with different Al nanodisk metasurfaces deposited onto a LED wafer and covered by a Nb_2_O_5_ layer. A schematic representation of a sample is shown in Figure 1(a). The LED wafer consists of several layers, including a sapphire substrate with a thickness of 240 μm, a 6 μm thick layer of GaN, 2 InGaN quantum wells with a thickness of 10 nm, a 75 nm thick layer of p-GaN, and a 20 nm thick layer of ITO. Each metasurface fabricated on the ITO layer is a square array of Al nanodisks with a height of 55 nm and a diameter of 100 nm. In total, 13 metasurfaces have been fabricated with different lattice constants (a) ranging from 180 nm to 280 nm, with a step size of 10 nm (for more details on the fabrication, see the Section 4). A Scanning Electron Microscope (SEM) image of a typical metasurface is shown in the SI (Figure S1(a)). As a final step, the Al metasurfaces were covered by a 215 nm layer of Nb_2_O_5_ layer to achieve an almost homogeneous environment of surrounding refractive index. The homogeneous environment enables more efficient diffraction by the Al array, leading to stronger SLR field distribution [45]. Due to the shadowing effect during the sputter deposition process, the surface of the Nb_2_O_5_ is not flat, as can be seen on the SEM image of the top surface shown in Figure 1(b). To study this shadowing effect, we have characterized the flatness of the deposited layer using atomic force microscopy (AFM). We notice that the deposited layer forms an ellipse-like shape on top of the Al nanodisks, as shown in Figure S1(b).

The Al metasurfaces are designed to tailor the emission from the MQWs by near-field coupling between the SLRs produced by the metasurfaces and the InGaN MQWs. For this reason, we optimized the dimensions of individual Al nanodisks embedded in the Nb_2_O_5_ layer on top of ITO/GaN to support localized surface plasmon resonances (LSPRs) and maximize the LSPRs at *λ* = 570 nm, which is the central wavelength of the InGaN MQWs. The optimal parameters of the Al nanodisk were found to be a height of 55 nm and a diameter of 100 nm. The calculated scattering cross-section (SCS) of an individual Al nanodisk with optimal dimensions is shown in Figure S2.

### 2.2 Directional emission with Al metasurfaces

**Figure 2:**
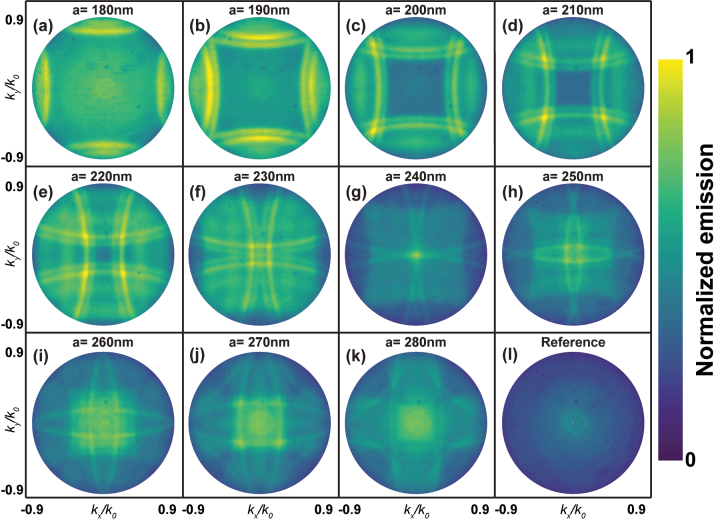
Backfocal plane images of the *λ* = 570 nm emission measured from metasurface samples covered by a layer of Nb_2_O_5_. The lattice constant of the corresponding metasurface varies from 180 nm (a) to 280 nm (k), with a step of 10 nm. The reference image (l) was taken from an area of the same wafer but without nanostructures. For all images, the *x*-axis is *k*_*x*_/*k*_0_ and the *y*-axis is *k*_*y*_/*k*_0_. The radius (0.9) of the BFP images corresponds to the NA of the collection objective. The data in each panel have been normalized such that the maximum PL in each plot is equal to unity.

Using Fourier microscopy, we have characterized the angular-resolved far-field emission *I*(*θ*, *ϕ*) from InGaN MQWs integrated with the Al metasurfaces. The experimental setup is described in the Section 4. The InGaN MQWs are excited by a continuous wave (CW) laser emitting at the wavelength of 405 nm and illuminating the sample through the sapphire side at normal incidence. The InGaN MQWs photoluminescence (PL) is distributed within the full solid angle [51]. However, the collected PL is limited by the numerical aperture (NA = 0.9) of the collection objective used in the experiment. The collected PL is sent to a spectrometer or a CCD camera. A bandpass filter in front of the CCD camera with a center wavelength of 570 nm and a transmission FWHM of 10 nm was used to filter the emission of the InGaN MQWs from the excitation laser. The CCD camera images the back focal plane (BFP) of the collection objective, which represents a map of the angle resolved far-field emission intensity *I*(*k*_*x*_, *k*_*y*_) in the coordinates of reciprocal space. These coordinates are *k*_*x*_ = *k*_0_ sin*θ* cos*ϕ* and *k*_*y*_ = *k*_0_ sin*θ* sin*ϕ* with *k*_0_ = 2*π*/*λ*, i.e., the wavevector in vacuum. In Figure 2, we plot BFP images of the emission PL pattern from metasurfaces with different lattice constants ranging from 180 nm (Figure 2(a)) to 280 nm (Figure 2(k)). These lattice constants are roughly half the emission wavelength of the MQWs, considering that the nanoparticles are embedded in Nb_2_O_5_ with a refractive index ∼2.3 in the visible.

The emission angles vary from 0° to 64°, which correspond to the NA of the collection objective. Each BFP image is normalized to its maximum and it has a 4-fold symmetry, as expected from the symmetry of the square lattice. The reference BFP image (Figure 2(i)) was taken from an area of the same sample including the Nb_2_O_5_ layer but without metasurfaces. The far-field emission of this reference sample has a Lambertian shape. For the metasurfaces samples, we observe a significant change in the far-field emission pattern compared to the reference as a result of near-field coupling between SLRs and the InGaN MQWs. The emission into defined directions is controlled by varying the lattice constant of the metasurfaces (as shown in Figure 2). In addition, we observe double bands in the BFP images (for example, Figure 2(c)). These bands are further discussed in Figure S4, and are associated with the slight mismatch in refractive indices between GaN (2.45) and Nb_2_O_5_ (2.34) at 570 nm. Al metasurfaces with *a* = 240 nm show that the emitted light can be beamed within a small solid angle, which has great potential for *μ*LEDS. Therefore, we have investigated this sample in more detail in the next sections.

### 2.3 Directional emission pattern

To investigate the origin of the directional emission pattern, we have explored the influence of the SLRs in the emission outcoupling. For a 2-dimensional array of particles, the diffractive orders are given by the grating equation:

(1) $$\pm k_{\text{diff}}=k_{\text{in}}+G_{l},$$

where ***k***_diff_ is the wave vector of the diffracted wave, ***k***_in_ is the wave vector of the incident wave and $G_{l}=\left(G_{x},G_{y}\right)=\left(\frac{2\pi }{a_{x}}p\hat{x},\frac{2\pi }{a_{y}}q\hat{y}\right)$ are the reciprocal lattice vectors with *a*_*x*_ and *a*_*y*_ the lattice constant in *x*-and *y*-directions, and *p* and *q* integers indicating the order of diffraction.

For the in-plane diffraction orders or Rayleigh anomalies (RAs), the out-of-plane component of the wave vector of the diffracted order (*k*_*z*_) equals 0. Using this condition and the grating equation relating the incident and diffractive waves, we obtain the dispersion relations of the Rayleigh Anomalies

(2) $$\begin{array}{ll} \nu_{RA}^{2} & ={\left(\frac{c_{0}}{n}\right)}^{2}\left[{k_{x}}^{2}+{k_{y}}^{2}+{\left(\frac{2\pi }{a_{x}}p\right)}^{2}+{\left(\frac{2\pi }{a_{y}}q\right)}^{2}\right.\\ & \;\left.+2k_{x}\frac{2\pi }{a_{x}}p+2k_{y}\frac{2\pi }{a_{y}}q\right], \end{array}$$

where $\nu_{\text{RA}}^{2}$ is the frequency of the RA with *c*_0_ the speed of light in vacuum, *n* the refractive index of the medium, *k*_*x*_ and *k*_*y*_ the wave vectors of the incident wave along the *x* and *y* directions.

**Figure 3:**
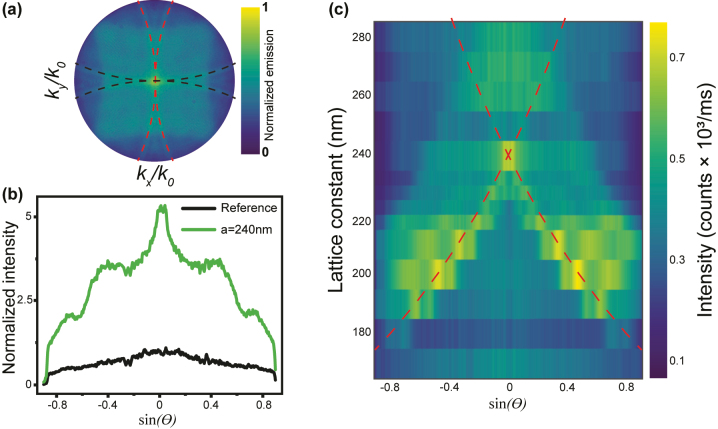
For Al metasurfaces with *a* = 240 nm. (a) BFP measurement (as in Figure 2). The dashed curves correspond to the RAs calculated with Eq. (2). The red and black dashed curves represent the diffraction orders [±1,0] and [0,±1], respectively. The BFP image has a radius of 0.9, which corresponds to the NA of the objective. (b) 2D-cut of the far-field emission intensity as a function of sin*θ*, with *θ* of the emission angle at *k*_*y*_ = 0. (c) Emission intensity as a function of the emission polar angle and lattice constant. The dashed red curves correspond to the [±1,0] diffraction orders.

We solve Eq. (2) for a given frequency (*ν*_RA_ = 525.952 THz, i.e., *λ* = 570 nm) and calculate the isofrequency dispersion curves for (*p*,*q*) = (±1,0), (0,±1) and (±1,±1), and different periodicities. The resulting isofrequency curves are plotted in Figure S3 with the red and black curves over the BFP measurements. The measurements and isofrequencies for *a* = 240 nm array are shown in Figure 3(a). The emission in the normal direction corresponds to the crossing of the (0,±1) and (±1,0) RAs at *k*_*x*_ = *k*_*y*_ = 0. Diffraction is thus responsible for the outcoupling of the emission trapped in the device.

### 2.4 Photoluminescence and beaming by the metasurfaces

To quantify the beaming within a small solid angle, we make a 2D plot of the far-field emission as a function of sin*θ* for Al metasurfaces with *a* = 240 nm, with *θ* ranging from −64° to 64° and *k*_*y*_ = 0, as shown in Figure 3(b). We obtain a PL emission enhancement factor of 5.2 within a small angle compared to the reference. To illustrate the dispersive behavior of the Al nanodisks with different lattice constants, we plot the far-field emission pattern over the emission angles as a function of the lattice constants, as shown in Figure 3(c). We observe an emission beaming at lower angles by increasing the lattice constant from 180 nm till the emission is mainly in the normal direction for *a* = 240 nm. In addition, we have fitted the far-field emission pattern with the solutions of Eq. (2), obtaining a good agreement with the measurements, as shown by dashed curves in Figure 3(c).

**Figure 4:**
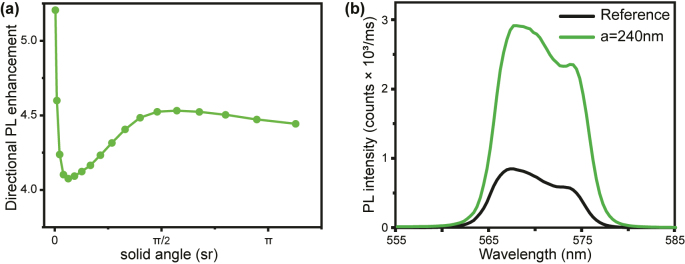
PL directional and emission enhancement. (a) Emission enhancement normalized to the reference as a function of the solid angle for the Al metasurfaces with *a* = 240 nm. (b) PL emission spectrum from the metasurface (green) and reference sample (black).

To quantitatively analyze the angular redistribution of the PL emission by the metasurfaces, we determine the directionality enhancement defined as the ratio of the emitted light intensity by the metasurfaces to the reference integrated over the solid angle. We convert from emitting polar angles to solid angles with:

(3) Ω=2π(1−cos(θ)),

where Ω is the solid angle (sr) and *θ* is the emitting polar angles (−64° to 64°) collected by the objective. In Figure 4(a), we plot the PL enhancement for the metasurfaces with *a* = 240 nm, observing a beaming enhancement of a factor around 5.2 for a narrow solid angle (representing an NA ≤0.1 ). This PL directionality enhancement confirms that SLRs represent an excellent approach for manipulating the emission from distant emitters and improving the outcoupling from InGaN MQWs. The beaming effect can be important for low-NA optical systems as they only collect light within narrow angles. In addition, we have measured the integrated PL spectrum over the NA of the objective from the sample with the Al metasurfaces (*a* = 240 nm) and the reference under the same experimental conditions. The integrated emission over the emission bandwidth (12 nm) with Al nanodisks is enhanced by a factor of 3.67 compared to the reference, as shown in Figure 4(b).

### 2.5 Time-resolved PL measurements

**Figure 5:**
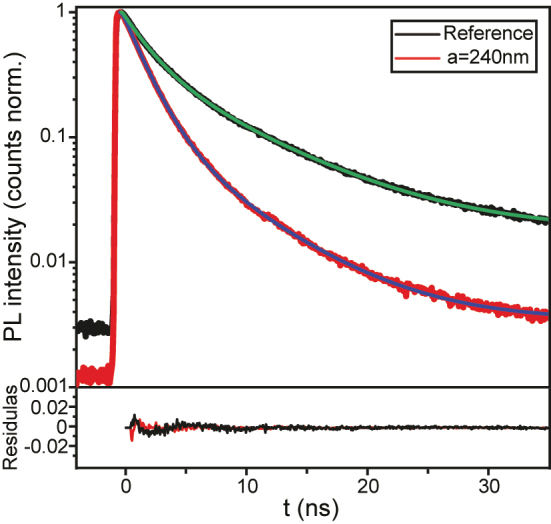
Time-resolved PL measurements. (a) PL decay curves of Al metasurfaces with *a* = 240 nm (red curve) and a reference without metasurfaces (black curve). The blue and green curves represent the bi-exponential fits to the Al metasurfaces and the reference measurements, respectively. The residuals of the fit are shown in the subpanel at the bottom.

We have investigated the influence of the near-field coupling on the emission decay rate from the MQWs by time-resolved PL (TRPL) measurements. These measurements are performed for the Al metasurfaces (with *a* = 240 nm) and the reference samples at room temperature. We have used the second-harmonic of a femtosecond Ti:Sapphire laser (*λ* = 405 nm) to excite the InGaN MQWs. The PL emission is directed to a time-correlated single-photon counting (TCSPC) detector to measure the histogram that defines the decay rate of the emission (see the Section 4). The TRPL response is analyzed based on the carrier rate equation in InGaN MQWs:

(4) $$-\frac{\text{d}N}{dt}=AN+BN^{2}+CN^{3},$$

where the *N* is the photon-excited carrier density. The coefficients *A* and *C* correspond to the nonradiative Shockley–Reed–Hall recombination rate and the Auger recombination rate, respectively. The coefficient *B* corresponds to the radiative recombination rate. The measurements have been performed using a low excitation power of 78 μW, hence Auger recombination (*C*) can be neglected. Figure 5 shows the measured normalized PL emission decays from the MQWs of the Al metasurface and the reference. These decays are fitted with a bi-exponential function to obtain the short-time (*τ*_*s*_) and long-time (*τ*_*l*_) exponential decays. The residuals from the fit, defined as the difference between the normalized measured data and the fit to those data, are shown in the lower panel of Figure 5. From the fits, we have determined the *τ*_*s*_ and *τ*_*l*_ to be 1.70 ± 0.06 ns and 6.10 ± 0.05 ns for the sample with the Al metasurfaces and 2.30 ± 0.04 ns and 7.10 ± 0.07 ns for the reference sample. These exponential decays are used to retrieve the radiative (*τ*_*r*_) and nonradiative (*τ*_*nr*_) recombination rates as described by Kim et al. in Ref. [52]:

(5) $$\tau_{r}=\frac{2\tau_{s}\tau_{l}}{\tau_{l}-\tau_{s}},$$

(6) $$\tau_{nr}=2\tau_{l}.$$

We have estimated *τ*_*r*_ and *τ*_*nr*_ to be 4.70 ± 0.2 ns and 12.20 ± 0.4 ns for the sample with the Al metasurfaces and 6.80 ± 0.14 ns and 14.20 ± 0.14 ns for the reference sample. The emission decay rates can be controlled by coupling the emitters to metallic nanoparticles, and this coupling can be controlled by the distance between the MQWs and the nanoparticle array [53]. Even though the large distance (95 nm) between the MQWs and the Al metasurfaces, we have obtained a shorter radiative recombination time from the MQWs emission in comparison with the reference due to the near-field coupling with the SLR. However, the Al metasurfaces introduce also Ohmic losses due to the metal absorption, resulting in a shorter nonradiative recombination time.

The internal quantum efficiency (IQE) can be calculated using *τ*_*r*_ and *τ*_*nr*_:

(7) $$\eta_{\text{IQE}}=\frac{\tau_{nr}}{\tau_{r}+\tau_{nr}},$$

which gives a value of *η*_IQE_ = 72.2 % for the emission of the sample with the Al metasurfaces, and of 67.6 % for the reference sample. Therefore, despite the Ohmic losses, the nanodisk array increases the emission efficiency of the MQWs.

### 2.6 Numerical simulations for Al metasurfaces

To investigate further the near-field coupling and the beaming of the emitted light, we have performed numerical simulations of the angular far-field emission intensity *I*(*θ*, *ϕ*) from the Al metasurfaces (with *a* = 240 nm) using the reciprocity principle and applying periodic boundary conditions (see Section 4) [54]. According to the reciprocity principle, the emission intensity *I*(*θ*, *ϕ*; **r**_1_) in the far-field along the direction (*θ*, *ϕ*) from a radiating dipole source **p**_1_ positioned at **r**_1_ can be calculated as [30, 55]:

(8) $$\begin{array}{ll} & I\left(\theta ,\phi ;r_{1}\right)\propto \overset{2\pi }{\underset{0}{\int }}\overset{\pi }{\underset{0}{\int }}\left[\underset{TE,TM}{\sum }|E\left(\theta ,\phi ,r_{1}\right)\right.\\ & \;\left.\times \cdot \;p_{1}\left(\theta_{p},\phi_{p},r_{1}\right){|}^{2}\right]\sin\theta_{p}d\theta_{p}d\phi_{p}, \end{array}$$

where **E**(*θ*, *ϕ*, **r**_1_) is the electric field, excited by the incident plane wave with *TE* and *TM* polarizations along the direction (*θ*, *ϕ*). The angles *θ*_*p*_ and *ϕ*_*p*_ are defining the dipole source **p**_1_ orientation. The integration is performed to average over the in-plane and out-plane distribution of the emitting dipole moments, and the emission of the dipoles is considered incoherent. The orientation of the emitting dipoles affects the far-field emission, so we determine this orientation using an experimental method as described in Ref. [56]. The determined orientation distribution is on average 55 % out-of-plane and 45 % in-plane, as shown in Figure S5. Therefore assuming that |**p**_1_| = 1, Eq. (8) can be written as:

(9) $$\begin{array}{ll} I\left(\theta ,\phi \right) & \propto \underset{TE,TM}{\sum }\underset{\text{MQWs}}{∭}\left[0.225\left(|E_{x}\left(\theta ,\phi ,r_{1}\right){|}^{2}\right.\right.\\ & \;\left.\left.+|E_{y}\left(\theta ,\phi ,r_{1}\right){|}^{2}\right)+0.55|E_{z}\left(\theta ,\phi ,r_{1}\right){|}^{2}\right]{\text{d}}^{3}r_{1}. \end{array}$$

**Figure 6:**
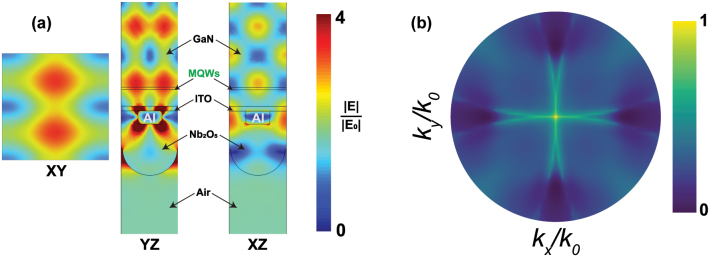
Numerical simulations for the fabricated Al metasurfaces. (a) Simulations of the spatial distribution of the electric field enhancement at *λ* = 570 nm and at *XY*, *YZ* and *XZ* planes. The *XY* plane corresponds to the center position of the MQWs. (b) Calculated back focal plane image at *λ* = 570 nm using reciprocity. The BFP image has a radius of 0.9, which corresponds to the NA of the objective in the setup.

From this last equation, the angular emission intensity *I*(*θ*, *ϕ*) is calculated by integrating over the volume occupied by the InGaN MQWs. Therefore, we use a finite element method (FEM) implemented in the COMSOL to simulate the plane wave excitation of the fabricated structure and calculate the electric field distribution $\left(E_{x}\left(r_{1}\right),E_{y}\left(r_{1}\right),E_{z}\left(r_{1}\right)\right)$ . To illustrate the near-field coupling, we have performed the numerical calculations for the Al metasurfaces with *a* = 240 nm based on the structural parameters obtained from SEM and AFM images (see Figure S1) and using a plane wave excitation incident in the normal direction (*θ* = 0°). Figure 6(a) shows the electric-field strength distribution normalized to the incident electric field (polarized along the *Y*-direction) in a unit cell for different planes: *XY*, *YZ* and *XY*. The *XZ* and *YZ* planes are defined through the center of the Al nanodisk, and *XY* is shown for the center position of the MQWs.

These fields distribution indicate that the SLR at 570 nm arises from the diffractive coupling between the Al nanoparticles in the array and it differs from the LSPRs, which are typically confined near the nanoparticles. The SLR field distribution reaches the position of the MQWs, which results in the modification of the far-field emission intensity and the consequent beaming of the generated light into a narrow solid angle. We have simulated the angular far-field emission (*I*(*k*_*x*_, *k*_*y*_)) using Eq. (9) by varying *θ* from 0° to 64° and *ϕ* range from 0° to 45° from the integrated electric fields over the MQWs volume. For the Al metasurface with *a* = 240 nm, we plot this simulation in Figure 6(b). Comparing these results with the BFP image of the metasurface (Figure 2(g)), we obtain an excellent agreement validating the reciprocity simulations and the interpretation of the role of SLRs in the definition of the directional emission. To simulate the pump enhancement provided by the Al metasurfaces, we have repeated the simulations using a plane wave excitation at normal incident with the wavelength of the pump laser (*λ* = 405 nm). We have calculated the electric field enhancement using Eq. (9) with and without metasurfaces over the occupied volume by MQWs. For the Al metasurfaces with *a* = 240 nm, the calculated pump enhancement is 1.45. Therefore, the emission enhancement measured from the MQWs coupled to the metasurface is the result of the combination of pump enhancement and directional outcoupling.

## 3 Conclusions

In summary, we have fabricated Al metasurfaces on top of a green LED wafer to beam and direct the emission from InGaN quantum wells. To illustrate the control of the angular emission pattern, we have measured the back focal plane images for the fabricated samples with different lattice constants. A photoluminescence enhancement of a factor 5.2 is obtained within a narrow solid angle with a simple square array of Al nanoparticles as a result of the near-field coupling between surface lattice resonance and the InGaN MQWs emission. We also observe a reduction of the exciton lifetime in the quantum wells due to both an increase in the radiative and nonradiative recombination rates. In particular, this proposed approach does not require etching the GaN layer, leading to the enhancement of the emission without introducing sidewall effects. Finally, numerical simulations prove that the enhancement in the emission, directionally, and the PL decay time is the result of the near-field coupling introduced by the surface lattice resonance. These results demonstrate that plasmonic metasurfaces can be integrated with commercial micro-LED structures to control the emission directionally and increase the emitted light intensity to achieve bright displays without external focusing components.

## 4 Methods

### 4.1 Finite element numerical simulation

We used COMSOL Multiphysics to simulate the Al metasurfaces using a three-dimensional model. The model is a unit cell (square lattice) with Floquet periodic boundary conditions on the horizontal edges reproducing the periodicity of the metasurfaces. The unit cell was excited using plane wave excitation through a port (IN) at the n-GaN side. The Floquet vector was defined by the corresponding wave vector projection of the incident plane wave. We simulated both polarizations (TE and TM) for the plane wave excitation along the (*θ*, *ϕ*) direction to retrieve the BFP image. In addition, we took into account the distribution of emitting dipole moment of 55 % out-of-plane and 45 % in-plane, as given in Eq. (9). To calculate the far-field emission, we integrate the distributed weighted field over the volume occupied by the MQWs. The optical constants used in the model were measured by ellipsometry.

### 4.2 Sample fabrication

Lumileds Holding B.V. fabricated the wafer. A metal-organic chemical vapor deposition (MOCVD) reactor was used to grow the GaN/InGaN layer on a sapphire substrate followed by thinning the substrate to 240 μm and polishing the backside. The grown layers consist of 2 InGaN/GaN quantum wells with a total thickness of 10 nm, a p-GaN layer with a thickness of 75 nm, and 6 μm GaN. More details of related materials and epitaxy technology are discussed in Refs. [57, 58]. A 20 nm thick layer of ITO covering the wafer as a conductive layer was deposited with RF-magnetron sputtering. To fabricate the Al metasurfaces, we used electron beam lithography (EBL) with a lift-off process. To prepare the sample for EBL exposure, a layer of polymethyl methacrylate (PMMA) positive resist with a thickness of 240 nm was spin-coated on top of the ITO. The PMMA was baked for 3 min at 135 °C, followed by 4 min at 185 °C. To nanopattern the PMMA with the nanodisks, we used an EBL system (EBL, Raith EBPG 5150, 100 kV). The exposed area for each lattice was 200 μm × 200 μm, and the lattice constant was varied from 180 nm to 280 nm with a step of 10 nm. After exposure, the PMMA was developed using MIBK/IPA 1:3 developer for 90 s, followed by 90 s in IPA to stop the development of PMMA. A layer of Al with a thickness of 55 nm was deposited using an electron beam evaporator. To remove the remaining PMMA, the sample was put in acetone for 4 h. Finally, a layer of Nb_2_O_5_ with a thickness of 405 nm was deposited using DC sputtering. To characterize the fabricated samples, we used scanning electron microscopy (SEM) and atomic force microscopy (AFM).

### 4.3 Fourier microscopy measurements

The fabricated samples were optically pumped using a continuous wave (CW) laser producing a collimated beam at a wavelength of 405 nm. Fourier microscopy was used to measure the angular resolved PL emission from the wafer with and without Al metasurfaces. The emitted PL was collected by 0.9NA Nikon Plan Fluor 100× and directed to a CCD camera (Andor Neo 5.5), which was used to measure the back focal plane (BFP) images. Before sending the emitted light to the CCD camera, the residual outgoing laser light was filtered out by a 405 nm notch filter, followed by a bandpass filter with a center wavelength of 570 nm.

### 4.4 Lifetime measurements

The lifetime measurements were performed in a Nikon Ti-E inverted microscope. The 810 nm output of a 80 MHz Ti:Sapph oscillator (MaiTai) was frequency doubled to 405 nm by a BBO crystal. The repetition rate of the laser was reduced to 5 MHz in a pulse picker (APE pulseSelect) and the laser power was reduced to 78 μW using optical density filters. The laser beam was focused on the sample through a 60X objective lens (CFI S Plan Fluor ELWD 60XC). The time-resolved emission was collected in an optical fiber and sent to a time correlated single photon counting (TCSPC) module (TimeHarp 300, Picoquant). The emission from the MQWs was collected after removing the reflected laser light with a 405 nm laser line filter and a 420 nm long pass filter. The data were analyzed with the SymPhoTime 64 software.

## Supplementary Material

Supplementary Material Details
