# Supplementary material for: Surface lattice resonances for beaming and outcoupling green μ LEDs emission
Source: Nanophotonics. 2023 Aug 4;12(18):3553–62. doi: 10.1515/nanoph-2023-0257 (PMC11501564; doi:10.1515/nanoph-2023-0257)
Supplement: Supplementary file 1 — Supplementary Material Details [file j_nanoph-2023-0257_suppl_001.pdf]

Research Article

Mohamed S. Abdelkhalik*, Aleksandr Vaskin, Toni López, Anton Matthijs Berghuis, Aimi Abass, and Jaime Gómez Rivas*

Supporting Information: Surface Lattice Resonances for Beaming and Outcoupling Green μLEDs Emission

https://doi.org/10.1515/sample-YYYY-XXXX
Received Month DD, YYYY; revised Month DD, YYYY; accepted Month DD, YYYY

This supplemental document contains the following information:

1. Scanning electron microscope for fabricated Al metasurfaces.
2. Scattering cross-section (SCS) calculations for individual Al nanodisk.
3. Calculation of diffraction orders dispersion for Al metasurfaces with different lattice constants.
4. Determination of the dipole emitting orientation.

1. Scanning electron microscope for fabricated Al metasurfaces

Before depositing the Nb_2O_5 layer, we characterized the fabricated Al metasurfaces on the top of the ITO layer using a Scanning electron microscope (SEM) to check the sample quality. Figure. S1 (a) shows an SEM image of the array with a= 240 nm. In addition, we characterized the same sample using Atomic Force Microscopy (AFM) after depositing the Nb_2O_5 layer. Figure. S1 (b) shows an AFM image of the same array.

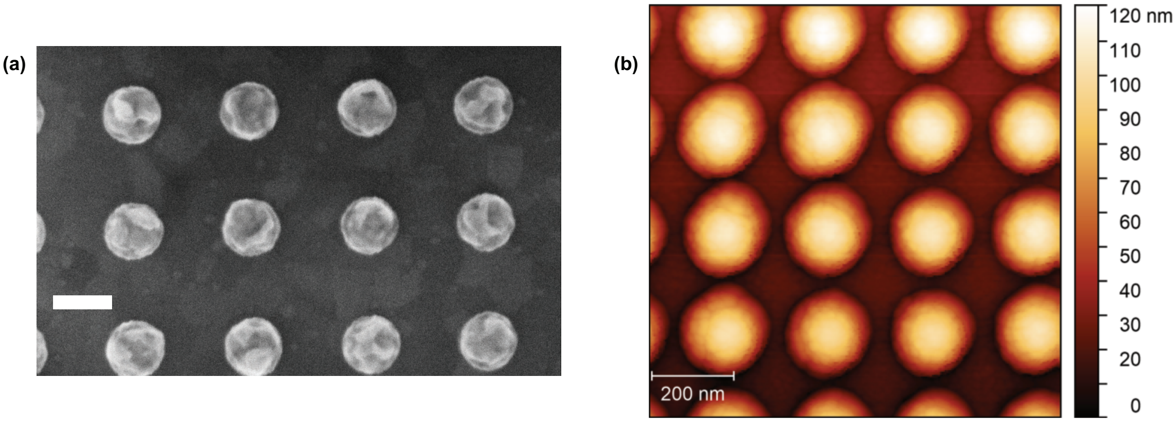

Fig. S1: (a) SEM image of the fabricated Al metasurfaces with a= 240 nm on top of ITO/p-GaN. The scale bar corresponds to 100 nm. (b) AFM of the Al nanodisks covered by Nb_2O_5.

***Corresponding author: Mohamed S. Abdelkhalik, Aleksandr Vaskin, Anton Matthijs Berghuis, Jaime Gómez Rivas,** Department of Applied Physics and Science Education, and Eindhoven Hendrik Casimir Institute, P.O. Box 513, 5600 MB Eindhoven, The Netherlands, e-mail: m.m.s.abdelkhalik.mohamed@tue.nl, J.Gomez.Rivas@tue.nl
Toni López, Aimi Abass, Lumileds Germany GmbH, D-52068 Aachen, Germany, e-mail: toni.lopez@lumileds.com

2. Scattering cross-section (SCS) calculations for individual Al nanodisk

We calculated the scattering cross section (SCS) for individual Al nanodisk with D=100 nm and h=55 nm, embedded in Nb_2O_5 layer on top ITO/GaN stack as a function of the incident wavelengths (nm).

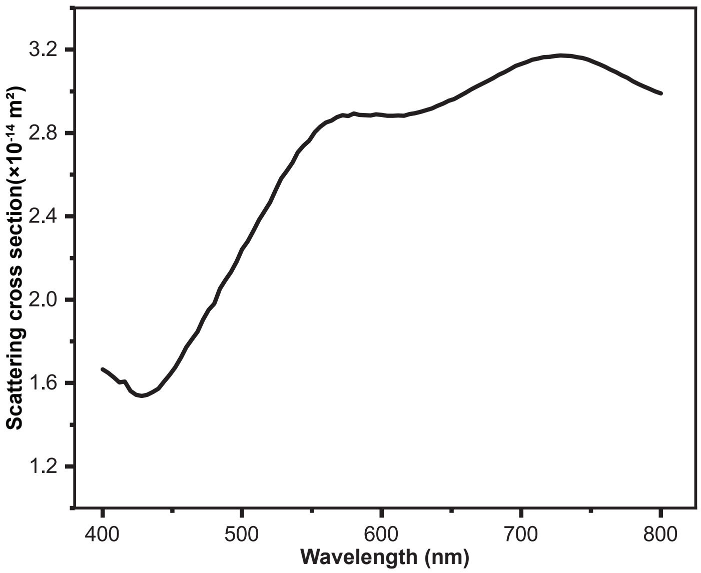

Fig. S2: Scattering cross-section (SCS) calculations for individual Al nanodisk with D=100 nm and h=55 nm

3. Calculation of diffraction orders dispersion for Al metasurfaces with different lattice constants

We here solved the diffraction equation at λ=570 nm for the in-plane diffraction orders (Eq. (2)) using the GaN refractive index of 2.45. The resulting dispersion (as shown in Fig. S3) for all measured lattice constants are plotted with the red, black and orange curves for (±1,0), (0,±1) and (±1,±1) diffraction orders, respectively. In addition, we solve the diffraction equation for the refractive index of GaN and Nb_2O_5 (n= 2.34) for the Al metasurfaces with a= 180 nm. We observe that the double dispersion curves correspond to the small mismatch in refractive index between GaN and Nb_2O_5 as shown in fig.S4.

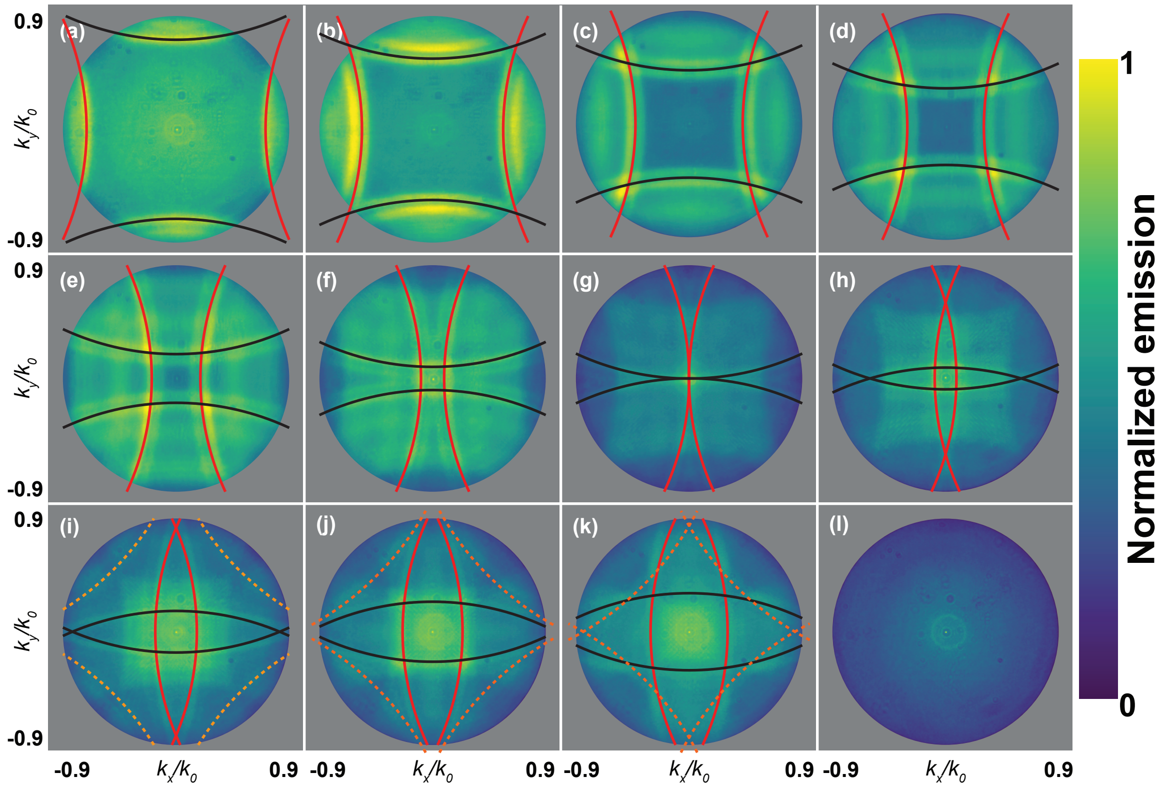

Fig. S3: Measured back focal plane images of the emission at λ=570 nm from the InGaN MQWs and metasurfaces with varying lattice constant $180\,$nm (a) to $280\,$nm (k) in steps of $10\,$nm. The reference image (l) was taken from an area of the same sample but without nanostructures. Red, black, and orange curves represent the inplane diffraction orders (0,$\pm$1), (0,$\pm$1), and ($\pm$1,$\pm$1), respectively. The BFP image has a radius of 0.9 which corresponds to the NA of the objective.

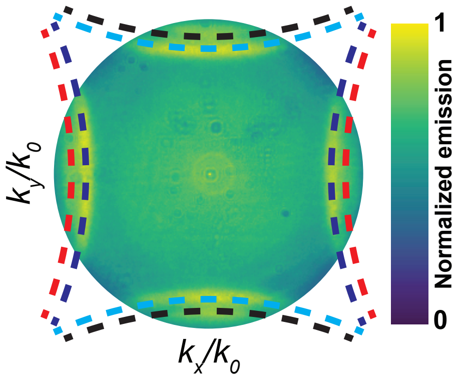

Fig. S4: Measured back focal plane images of the emission at λ=570 nm from the InGaN MQWs and metasurfaces with a lattice constant of $180\,$nm. Red and black curves represent the diffraction orders (0,$\pm$1), (0,$\pm$1) for the GaN refractive index. Blue and sky blue lines represent the diffraction orders (0,$\pm$1), (0,$\pm$1) for Nb_2O_5 refractive index. The BFP image has a radius of 0.9 which corresponds to the NA of the objective.

4. Determination of the dipole emitting orientation

To calculate the orientation of the emitting dipole moments, we excited the sample with a 405 nm laser and measured the sidewalls emission from InGaN MQWs changing the polarization for the collected light from TE to TM[1]. For both polarizations, we integrated the collected emission from 550 nm to 650 nm and divided it by the total unpolarized collected light. The calculated ratio is on average 45% in-plane (TE) and 55% out-of-plane (TM).

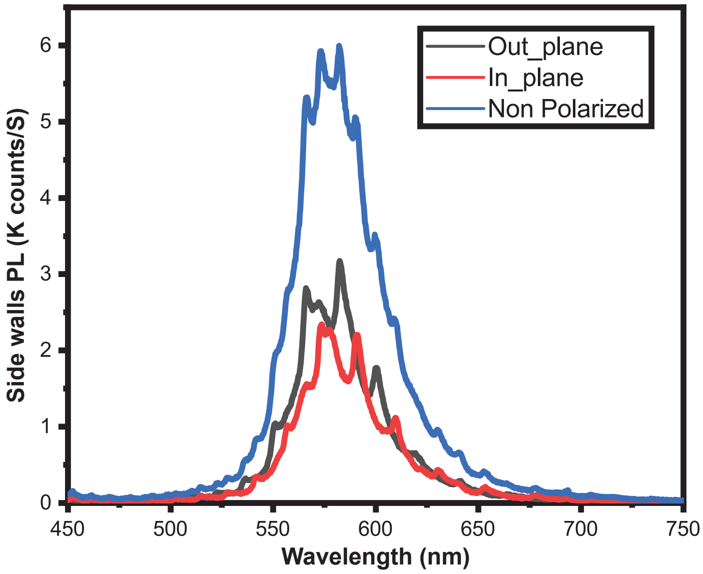

Fig. S5: Measured sidewalls emission from InGaN MQWs using a 405 nm excitation laser. Red, black, and sky blue curves represent the in-plane, out-plane, and total collected emission.

References

[1] H. Masui, N. N. Fellows, S. Nakamura, and S. P. DenBaars, "Optical polarization characteristics of light emission from sidewalls of primary-color light-emitting diodes," *Semiconductor Science and Technology*, vol. 23, 2008.
